# Association between regulator inspection and ratings on primary care prescribing: an observational study in England 2014 to 2019

**DOI:** 10.1186/s12913-024-10906-3

**Published:** 2024-05-29

**Authors:** Thomas Allen, Kieran Walshe, Nathan Proudlove, Matt Sutton

**Affiliations:** 1 Manchester Centre for Health Economics, University of Manchester, 4.305 Jean McFarlane Building, Oxford Road, M13 9PL Manchester, UK; 2 Alliance Manchester Business School, University of Manchester, M15 6PB Manchester, UK; 3 Health Organisation, Policy and Economics, University of Manchester, M13 9PL Manchester, UK; 4 Danish Centre for Health Economics, University of Southern Denmark, Odense, Denmark

**Keywords:** Inappropriate prescribing, Primary health care, Quality assurance, Health care, Data analysis, General practice standards

## Abstract

**Background:**

Healthcare regulators in many countries undertake inspections of healthcare providers and publish inspection outcomes with the intention of improving quality of care. Comprehensive inspections of general practices in England by the Care Quality Commission began for the first time in 2014. It is assumed that inspection and rating will raise standards and improve care, but the presence and extent of any improvements is unknown. We aim to determine if practice inspection ratings are associated with past performance on prescribing indicators and if prescribing behaviour changes following inspection.

**Methods:**

Longitudinal study using a dataset of 6771 general practices in England. Practice inspection date and score was linked with monthly practice-level data on prescribing indicators relating to antibiotics, hypnotics and non-steroidal anti-inflammatory drugs. The sample covers practices receiving their first inspection between September 2014 and December 2018. Regression analysis and the differential timing of inspections is used to identify the impact on prescribing.

**Results:**

Better-rated practices had better prescribing in the period before inspections began. In the six months following inspections, no overall change in prescribing was observed. However, the differences between the best and worse rated practices were reduced but not fully. The same is also true when taking a longer-term view. There is little evidence that practices responded in anticipation of inspection or reacted differently once the ratings were made public.

**Conclusion:**

While some of the observed historic variation in prescribing behaviour has been lessened by the process of inspection and ratings, we find this change is small and appears to come from both improvements among lower-rated practices and deteriorations among higher-rated practices. While inspection and rating no doubt had other impacts, these prescribing indicators were largely unchanged.

**Supplementary Information:**

The online version contains supplementary material available at 10.1186/s12913-024-10906-3.

## Background

Healthcare regulators in many countries undertake inspections of healthcare providers and publish the outcomes of those inspections in some form, with the intention of promoting compliance with inspection standards or other guidance and so improving the quality of care [1]. Despite the widespread use of such inspections, their mechanism of action and their impact is poorly understood and often contentious [2–4].Furthermore, most research has focused on secondary care and recent systematic review highlights the lack of knowledge on the impact of inspection in primary care, both internationally and in the UK in particular [5].

In England, high-profile failures in the provision of care in some hospitals led to a public inquiry and several critical reports by government and parliament [6–10].This attention resulted in questions being raised about the ability of the healthcare regulator, the Care Quality Commission (CQC), to adequately fulfil its role to monitor and oversee the quality of health and social care. A new CQC regulation model was developed in response to these concerns and the CQC began to implement this model in 2014 [11].

The new model included a programme of extensive inspection and rating of all general practices in England, approximately 7000 practices. The first practices were inspected in September 2014 and had their inspection ratings published in November 2014. When inspecting practices, the CQC focused on five key domains of care: Safe, Effective, Responsive, Caring and Well-led plus an additional Overall rating, which aggregates the ratings from each domain. On each of these six, a rating of ‘Outstanding’, ‘Good’, ‘Requires Improvement’ or ‘Inadequate’ was awarded. The inspection ratings received must be displayed by the practice in an area visible to patients.

In addition to inspecting and rating practices, the CQC also monitored practice performance using a range of performance measures under its Intelligent Monitoring system [12–14]. For general practices, this monitoring included four indicators relating to the prescribing behaviour of practices. These four indicators targeted the prescribing of antibiotics, hypnotics and non-steroidal anti-inflammatory drugs due to concerns about patient safety from over-prescribing of these drugs. The importance of appropriate antibiotic prescribing has been highlighted by the Chief Medical Officer [15] and by the United Nations [16]. Overuse of hypnotics is linked to higher mortality [17], while overuse of selected non-steroidal anti-inflammatory drugs is linked to greater cardiovascular risk [18].

The relationship between CQC inspection and performance has been investigated in secondary care but not in primary care [19, 20]. Our aim was to determine if practice rating scores were associated with past performance on these four prescribing indicators and also if prescribing behaviour changed following the inspection.

## Methods

### Data

To analyse the response of practice prescribing to healthcare regulation we used two sources of data: (1) CQC inspection and ratings data [21] and (2) general practice prescribing data [22]. We also controlled for practice population size [23].

CQC inspection and ratings data were provided by the CQC. These data included the inspection date, rating publication date and the inspection rating score for general practices which received their first inspection between September 2014 and December 2018. The Overall rating is used throughout this study as it represents how the practice performance was assessed over a range of areas, giving the most complete indication of quality.

General practices prescribing data contained monthly prescribing data for all practices in England for the period April 2013 to June 2019, detailing the total number of items for each medicine prescribed by the practice in each month [22].

The following indicators were generated from the prescribing data:

1. **Total number of antibacterial drug items per 100 Specific-Therapeutic-Group-Age-sex weightings-Related Prescribing-Units (STAR-PUs).** STAR-PUs were used to adjust for those practices expected to use more of a certain drug type due to differences in demographics, usually due to an older population [24, 25]. Overuse of antibiotics can result in antibacterial resistance. They should only be used when appropriate to maintain their effectiveness. Practices which prescribe a large number of antibacterial drugs may be using them inappropriately. A higher value on this indicator may suggest poor prescribing behaviour.
2. **Broad-spectrum antibiotics as a percentage of all antibiotics.** Narrow-spectrum antibiotics are often cheaper and effective against specific bacterial infections. They should be used instead of broad-spectrum antibiotics, unless they are known to be ineffective against the target bacteria. A higher value on this indicator suggests poor prescribing behaviour.
3. **Total number of hypnotic drug items per 1000 STAR-PUs.** When used for long periods of times these drugs have a high risk of side effects. Their use should be restricted to only appropriate cases. A higher value on this indicator may suggest poor prescribing behaviour.
4. **Percentage of Non-Steroidal Anti-Inflammatory Drugs (NSAIDs) that are Ibuprofen and Naproxen.** The long-term use of this drug group has been linked to cardiovascular and gastro-intestinal events. Risks are lower for Ibuprofen and Naproxen and therefore their use should be greater. A lower value on this indicator suggests poor prescribing behaviour.

These prescribing indicators were uniquely suitable for our analysis as they were used in the CQC Intelligent Monitoring framework as well as being monitored under the NHS Key Therapeutic Topics [12, 26]. Furthermore, while CQC Intelligent Monitoring included 33 indicators in total, only these four prescribing indicators are generated from data that is freely available at a monthly frequency that would allow the impact of inspection to be investigated.

The data from these sources were linked to form a single dataset on inspections, ratings, prescribing indicators and practice population size for 6990 practices. Data were cleaned to remove 180 practices which closed between April 2013 and June 2019. Additionally, 39 very small practices with fewer than 1000 patients were excluded as practices observed to have fewer than 1000 patients are typically either serving specific populations (such as people experiencing homelessness) or are in the process of closing, opening, or merging. The final analysis sample consisted of 6771 practices.

#### Analytical approach

First, we assessed the degree to which such prescribing behaviours differed according to subsequent ratings before the inspection cycle began. This would indicate if practice ratings captured either prescribing behaviour directly or other aspects of practice quality that influenced prescribing behaviour. Using data from April 2013 to August 2014, before the earliest inspections, we estimated the association between performance on each prescribing indicator and the inspection rating the practice would go on to receive. We controlled for practice size and prescribing trends, the latter using a set of 17 categorical variables for the sequence of months. A panel data random effects model was used to allow for repeated observations. Where the indicator represented a percentage, the regressions are weighted by the denominator.

Second, we assess if prescribing behaviours changed after inspection. As there was a lag between the inspection visit and the rating being published, changes in prescribing behaviour may have been prompted by the inspection visit, the published inspection rating or both. We hypothesised the strongest effect was likely to follow the inspection visit, as this was the intervention most noticeable to practices. We further hypothesised that changes would differ between practices that received different inspection ratings.

We estimated three specifications, all using a linear fixed effects model, and all using data from April 2013 to June 2019. As practices were inspected at different times throughout 2014–2017, each specification takes advantage of this staggered inspection schedule. Practices act as a control group either before they are ever inspected or outside of the specific inspection period modelled in each specification. Regressions included indicators for month-year and for practice, and practice population size.

Our first specification modelled performance on each prescribing indicator in the six months post-inspection and did not differentiate any changes by inspection rating scores. This provided the overall impact of practice inspection on the selected prescribing indicators.

Our second specification modelled performance on each prescribing indicator by inspection rating score. The inspection rating score is interacted with the post-inspection period covering the inspection month and the six months immediately following. This provided the post-inspection change for each inspection rating score.

Our third specification included the inspection rating score interacted with the month prior to inspection and with the months more than six months post-inspection. This provided any impact in anticipation of inspection and any longer-term impacts on each prescribing indicator.

All model equations and variables are described fully in the online appendix. In additional models we replaced the inspection date with the date the rating was published, typically three months after the inspection. All data analysis were conducted with Stata Version 16.

## Results

The majority of practices were rated as Good (79.56%), followed by Requires Improvement (12.94%), Outstanding (4.3%) and Inadequate (3.2%).

Summary statistics for prescribing indicators and practice size in the pre-inspection period are shown in Table 1. On average, practices issued 11.59 antibacterial prescriptions per month per 100 STARPUs and 5.5% of antibiotics prescribed were broad spectrum. Practices issued on average 5.55 hypnotic prescriptions per month per 1000 STARPUs. Of all non-steroidal anti-inflammatory drugs, 67.60% were Ibuprofen/Naproxen. Finally, the average size of the practice population during this period was 7533 patients.

**Table 1:** Summary statistics for prescribing indicators and registered practice population

Indicator name	Mean	SD	5th percentile	95th percentile
Antibacterial prescriptions per 100 STAR-PUs	11.59	5.05	7.00	16.31
Percentage of broad spectrum antibiotics	5.50	3.09	1.65	11.19
Hypnotic prescriptions per 1000 STAR-PUs	5.55	3.78	1.73	11.67
Percentage of NSAIDs that are Ibuprofen/Naproxen	67.60	12.70	44.54	86.27
Registered practice population	7,533	4,312	2,267	15,072

Note: values are averaged over the pre-inspection period: April 2013 to September 2014

**Fig. 1:**
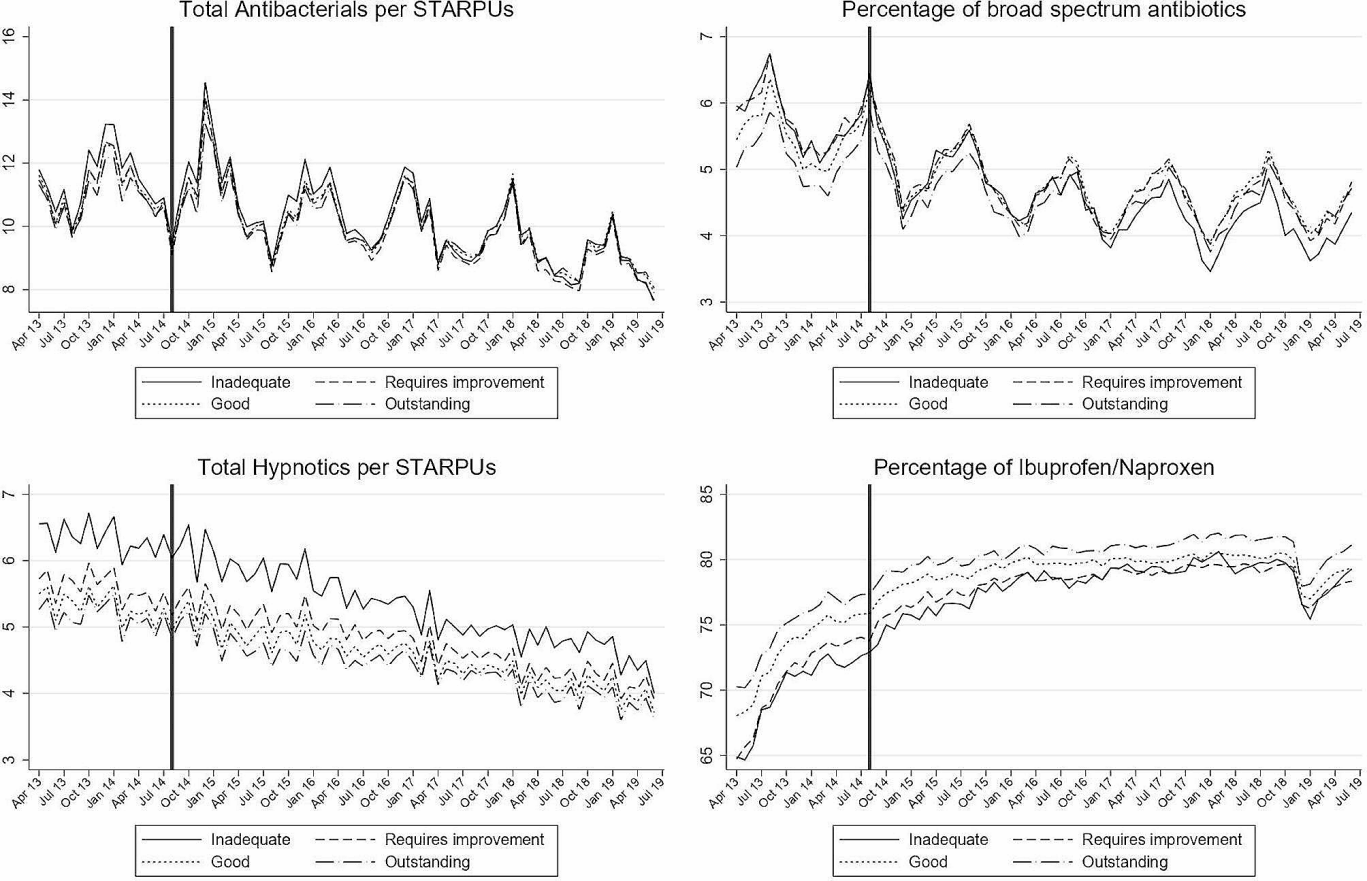
Mean prescribing indicator performance by rating score 2013 to 2019. Note: Solid line at September 2014 marks the start of the inspection regime

Across all indicators there was evidence of variation in prescribing behaviour according to practice inspection rating score (Fig. 1; Table 2). These differences were in the expected direction, with poorer prescribing behaviour found in practices with worse inspection rating scores. For example, prior to inspection, practices with an Outstanding inspection rating score were prescribing fewer broad spectrum antibiotics and a greater proportion of Ibuprofen/Naproxen.

**Table 2:** Differences in prescribing behaviour before the inspection month

	Antibacterial prescriptions per 100 STAR-PUs	Percentage of broad spectrum antibiotics	Hypnotic prescriptions per 1000 STAR-PUs	Percentage of NSAIDs that are Ibuprofen/Naproxen
Inadequate	0.0866 [-0.327,0.500]	0.278 [-0.113,0.668]	0.862^**^ [0.282,1.443]	-3.374^***^ [-4.837,-1.911]
Requires Improvement	-0.193 [-0.440,0.054]	0.370^***^ [0.179,0.561]	0.198 [-0.051,0.447]	-2.966^***^ [-3.701,-2.231]
Good	Reference category	Reference category	Reference category	Reference category
Outstanding	-0.00851 [-0.310,0.293]	-0.251 [-0.530,0.027]	-0.0100 [-0.450,0.430]	1.813^***^ [0.751,2.875]
Observations	115,088	113,790	114,918	115,071
Practices	6,771	6,771	6,771	6,771
R^2^ (within)	0.284	0.0782	0.0664	0.315

Reference category: Good rating. Random effects regression. Robust 95% confidence intervals in parentheses ^*^p < 0.05, ^**^p < 0.01, ^***^p < 0.001

Month dummies included for pre-inspection period April 2013 to August 2014. Model controls for practice population size, percentage indicators weighted by denominator

From Table 2, practices that, when later inspected, received ratings of Requires Improvement or Inadequate generally had worse prescribing behaviour. Of the eight coefficients relating to these ratings, seven suggested worse prescribing behaviour, four of which were statistically significant at *p* < 0.05 or better. The largest effect was found on the percentage of Ibuprofen/Naproxen, where the coefficient for Inadequate practices suggests these prescribe 3.374% points fewer of the (more desirable) non-steroidal anti-inflammatory drugs [95% CI: -4.837,-1.911]. This difference represented 25% of the standard deviation for this indicator. In contrast, practices with an Outstanding inspection rating tended to perform better than those rated Good. The signs of all four coefficients suggested better prescribing, although only the coefficient on percentage of Ibuprofen/Naproxen was statistically significant.

Table 3 presents results from four regressions, estimating the change in four indicators of prescribing performance in the six months following an inspection. These estimates combine practices receiving all four inspection rating scores and overall these is no statistically significant change.

**Table 3:** Changes in prescribing behaviour post-inspection

	Antibacterial prescriptions per 100 STAR-PUs	Percentage of broad spectrum antibiotics	Hypnotic prescriptions per 1000 STAR-PUs	Percentage of NSAIDs that are Ibuprofen/Naproxen
Six months post inspection	0.0634 [-0.025,0.152]	0.0333 [-0.015,0.081]	-0.00755 [-0.048,0.033]	-0.0294 [-0.184,0.125]
Observations	276,032	272,210	275,520	275,973
Practices	6,771	6,771	6,771	6,771
R^2^	0.790	0.657	0.900	0.814

Least-squares dummy variable model. Robust 95% confidence intervals in parentheses, clustered by practice ^*^p < 0.05, ^**^p < 0.01, ^***^p < 0.001 Month dummies included for April 2013 to June 2019. Model controls for practice population size, percentage indicators weighted by denominator

From Table 4 we observe that practices with poorer inspection ratings (Inadequate or Requires Improvement) improved their prescribing after inspection, while practices with better inspection ratings (Good and Outstanding) worsened. These changes are statistically significant for antibacterial prescriptions in Outstanding practices; for broad spectrum antibiotics in Requires Improvement and Good practices; and for NSAIDs in Requires Improvement practices. For example, in this post-inspection period, practices with a Requires Improvement rating increased their prescribing of Ibuprofen/Naproxen by 0.772% points [95% CI: 0.401,1.143]. This is an improvement in prescribing representing 6% of the standard deviation for this indicator.

**Table 4:** Changes in prescribing behaviour post-inspection by rating score

	Antibacterial prescriptions per 100 STAR-PUs	Percentage of broad spectrum antibiotics	Hypnotic prescriptions per 1000 STAR-PUs	Percentage of NSAIDs that are Ibuprofen/Naproxen
**Six months post inspection interacted with**:				
Inadequate	-0.115 [-0.368,0.139]	-0.151 [-0.360,0.059]	-0.121 [-0.302,0.059]	0.502 [-0.241,1.245]
Requires Improvement	0.0332 [-0.109,0.176]	-0.139^*^ [-0.250,-0.028]	-0.0667 [-0.151,0.018]	0.772^***^ [0.401,1.143]
Good	0.0670 [-0.017,0.151]	0.0567^*^ [0.005,0.108]	0.00326 [-0.040,0.047]	-0.131 [-0.297,0.034]
Outstanding	0.221^*^ [0.031,0.410]	0.124 [-0.048,0.296]	0.0496 [-0.133,0.232]	-0.447 [-0.963,0.069]
Observations	276,032	272,210	275,520	275,973
Practices	6,771	6,771	6,771	6,771
R^2^	0.790	0.657	0.900	0.814

Least-squares dummy variable model. Robust 95% confidence intervals in parentheses, clustered by practice ^*^p < 0.05, ^**^p < 0.01, ^***^p < 0.001 Month dummies included for April 2013 to June 2019. Model controls for practice population size, percentage indicators weighed by denominator

Changes in prescribing behaviour in anticipation of an inspection were statistically significant only for practices rated Requires Improvement and only relating to NSAIDs (Table 5). Changes in prescribing behaviour also appear to exist beyond the six-month period previously measured. The sign on all coefficients for Inadequate and Requires Improvement practices suggest improved prescribing in the longer term and six of these eight coefficients are statistically significant at *p* < 0.05 or better. The sign on all coefficients for Good and Outstanding practices suggest worsening prescribing in the longer term and five of these eight coefficients are statistically significant at *p* < 0.05 or better.

In supplementary analysis using the date of publication of the rating as the intervention point of interest, we found no substantive differences in how prescribing behaviour changes when compared with the analysis using inspection date presented above.

**Table 5:** Changes in prescribing behaviour pre- and post-inspection by rating score

	Antibacterial prescriptions per 100 STAR-PUs	Percentage of broad spectrum antibiotics	Hypnotic prescriptions per 1000 STAR-PUs	Percentage of NSAIDs that are Ibuprofen/Naproxen
**One month pre inspection interacted with**:				
Inadequate	-0.0738 [-0.344,0.196]	-0.0248 [-0.274,0.225]	-0.00974 [-0.184,0.164]	0.636 [-0.193,1.465]
Requires Improvement	0.00374 [-0.117,0.125]	-0.0387 [-0.178,0.101]	-0.0601 [-0.149,0.0285]	0.560^**^ [0.176,0.944]
Good	0.0161 [-0.031,0.063]	0.0445 [-0.001,0.089]	0.0115 [-0.025,0.048]	-0.0574 [-0.183,0.068]
Outstanding	0.0882 [-0.084,0.260]	0.169 [-0.022,0.360]	0.0464 [-0.155,0.248]	-0.186 [-0.748,0.375]
**Six months post inspection interacted with**:				
Inadequate	-0.166 [-0.411,0.078]	-0.162 [-0.370,0.045]	-0.110 [-0.295,0.074]	0.588 [-0.185,1.360]
Requires Improvement	-0.0155 [-0.127,0.097]	-0.154^**^ [-0.264,-0.044]	-0.0745 [-0.160,0.011]	0.786^***^ [0.411,1.161]
Good	0.0336 [-0.018,0.085]	0.0402 [-0.002,0.083]	0.0131 [-0.027,0.053]	-0.112 [-0.243,0.0179]
Outstanding	0.177^*^ [0.004,0.350]	0.110 [-0.074,0.294]	0.0389 [-0.163,0.241]	-0.372 [-0.910,0.166]
**Remaining months post inspection interacted with**:				
Inadequate	-0.331^**^ [-0.581,-0.080]	-0.354^**^ [-0.615,-0.094]	-0.413^**^ [-0.712,-0.114]	1.286^*^ [0.227,2.346]
Requires Improvement	-0.0786 [-0.225,0.068]	-0.169^*^ [-0.306,-0.033]	-0.0740 [-0.183,0.035]	1.055^***^ [0.547,1.562]
Good	0.109^*^ [0.0100,0.209]	0.113^***^ [0.049,0.176]	0.0382 [-0.021,0.097]	-0.291^**^ [-0.502,-0.080]
Outstanding	0.296^**^ [0.100,0.492]	0.273^**^ [0.077,0.468]	0.0906 [-0.102,0.284]	-0.455 [-1.188,0.278]
Observations	507,633	499,980	506,610	507,533
Practices	6771	6771	6771	6771
R^2^	0.775	0.591	0.868	0.750

Least-squares dummy variable model. Robust 95% confidence intervals in parentheses, clustered by practice ^*^p < 0.05, ^**^p < 0.01, ^***^p < 0.001 Month dummies included for April 2013 to June 2019. Model controls for practice population size, percentage indicators weighted by denominator

## Discussion

### Summary

Prior to inspection, we found statistically significant differences in prescribing indicators according to subsequent rating category. These differences followed the expected pattern, with better prescribing behaviour being associated with better ratings scores.

There was no overall impact of inspection in the six months after practices were inspected when grouping together all inspection rating scores. Prescribing behaviour generally improved for practices rated Inadequate or Requires Improvement, but worsened for those rated Good or Outstanding. In the longer term these different responses to inspection grew and reduce pre-existing variation in prescribing performance by rating.

### Strengths and limitations

This was the first study to measure the impact of the comprehensive CQC inspection system in primary care. The prescribing data used was collected for all practices and published at monthly intervals, providing a rich source of information about prescribing behaviour. We controlled for practice size and used panel data methods to control for unobservable differences between practices that could affect their prescribing.

However, our analysis was also limited in its focus on only four prescribing indicators. This was arguably a narrow lens through which to determine practice response to inspection, although these indicators were monitored by the CQC. That most practices were rated as Good is also a limitation for our study, suggesting that many practices may not have been motivated to change or improve.

### Comparison with existing literature

External inspections of this type, certainly have the potential to change behaviour and do so via influencing various mediators of organisational change [5]. Similar analysis has also looked at the association between CQC inspections and relevant indicators in hospital maternity and emergency departments [19, 20]. These studies found no association between inspection scores and performance indicators, both when looking at pre- or post-inspection performance. Our findings in primary care depart from this literature slightly in two ways. Firstly, we found modest associations between inspection ratings scores and prior performance. Secondly, we found some small changes post-inspection for practices with different ratings. However, as the post-inspection changes differed by the rating received, they effectively cancelled each other out. This resulted in finding no overall association between inspection and these indicators.

There was also no positive, clinically significant impact on adverse events following hospital inspection [27]. Other interventions to targeting prescribing, such as regulatory risk communications [28], have been more successful in changing behaviour.

## Conclusion

Prescribing behaviour, particularly that of antibacterial drugs, is a global issue and healthcare regulation may be one method by which to promote appropriate behaviour. There is some evidence that data on prescribing behaviour was modestly predictive of subsequent inspection ratings for general practices, but it does not seem likely that this relationship would be sufficiently strong to be used in making judgements on whether, when and where to inspect in general practice. This raises some cautions about the capacity to be more responsive and intelligence-led in the inspection programme. While some of the observed historic variation in prescribing behaviour has been reduced by the process of CQC inspection and ratings. This change was small and came from better practices worsening as well as worse practices improving, such that overall prescribing was unchanged by inspection. Though the lack of improvement following inspection might be concerning, especially given the resources dedicated to inspection, research on a wider range of performance measures would be needed before the value of inspection could be determined.

### Electronic supplementary material

Below is the link to the electronic supplementary material.

Supplementary Material 1

## Data Availability

The datasets used and/or analysed during the current study are available from the corresponding author on reasonable request.
