# Supplementary material for: Association between regulator inspection and ratings on primary care prescribing: an observational study in England 2014 to 2019
Source: BMC Health Serv Res. 2024 May 29;24:679. doi: 10.1186/s12913-024-10906-3 (PMC11137981; doi:10.1186/s12913-024-10906-3)
Supplement: Supplementary file 1 — Supplementary Material 1 [file 12913_2024_10906_MOESM1_ESM.docx]

**Impact of regulator inspection and ratings on primary care prescribing. An observational study in England 2014 to 2019**

Thomas Allen^1^, Kieran Walshe^2^, Nathan Proudlove^2^, Matt Sutton^3^

^1^ Manchester Centre for Health Economics, University of Manchester, 4.305 Jean McFarlane Building, Oxford Road, Manchester, M13 9PL, UK. [thomas.allen@manchester.ac.uk](mailto:thomas.allen@manchester.ac.uk)

^2^ Alliance Manchester Business School, University of Manchester, Manchester, M15 6PB, UK

^3^ Health Organisation, Policy and Economics, University of Manchester, University of Manchester, Manchester, M13 9PL, UK

**Regression equations**

The extent to which indicator behaviour differs by subsequent rating score can be assessed with Equation 1.

|  | $y_{it}=b_{0}+{\sum_{k=1}^{4} {b_{2k}[r}_{i}^{k}]+b}_{3}{size}_{it}+\tau_{t}+a_{i}+e_{it}$ | 1 |
| --- | --- | --- |

Where $y$ is the prescribing indicator of interest measured for practice $i$ in month $t$. $r$ is a set of four categorical variables taking the value 1 for practices with each rating such that $r_{i}^{1}$ = 1 for ‘Inadequate’, $r_{i}^{2}$ = 1 for ‘Requires Improvement’, $r_{i}^{3}$ = 1 for ‘Good’ and $r_{i}^{4}$= 1 for ‘Outstanding’. Differences observed in the coefficients $b_{2k}$ would suggest differences in indicators by rating score, but before the inspection cycle began. $size$ is a continuous variable measuring the practice population size. $\tau$ is a set of categorical variables, one for each month of data, to capture macro changes in indicators over time. This equation is estimated over the 17 months between the start of our data in April 2013 and the first practice inspections in September 2014. The variable $a$ is a set of practice effects and $e$ is an idiosyncratic error term. Equation 1 is estimated using a Random Effects model to allow for the effect of practice rating, which is fixed, to be estimated. Where the indicator is represented as a percentage (two and four in the list above) the regressions are weighted by the denominator.

We estimate three specifications, all using a least-squares dummy variable model, and where the indicator is represented as a percentage, weighting regressions by the denominator.

Our first specification is shown in Equation 2.

| $y_{it}=b_{0}+b_{1}{post\_1}_{it}+{b_{2}{size}_{it}+\tau}_{t}+a_{i}+e_{it}$ | 2 |
| --- | --- |

Where $y$, $size$, $\tau, a$, and $e$ are defined as in Equation 1. In this specification the post inspection changes in prescribing, measured by ${post\_1}_{it}$, are averaged across all rating scores. This provides the overall effect of practice inspection on the selected prescribing indicators.

Our second specification is shown in Equation 3.

| $y_{it}=b_{0}+\sum_{k=1}^{4} {b_{1k}[post\_1}_{it}^{k}]+{b_{2}{size}_{it}+\tau}_{t}+a_{i}+e_{it}$ | 3 |
| --- | --- |

Where $y$, $size$, $\tau, a$, and $e$ are defined as in Equation 1. ${post\_1}_{it}^{k}$ represent four different variables defining the post inspection time period for each of the four rating scores. The post inspection period covers the inspection month and the six months immediately following. This period differs by practice as practices are inspected at different times throughout 2014 – 2017. The four coefficients, $b_{1k}$, measure the change in prescribing indicator, $y_{it}$, occurring in these post inspection periods when compared to practices not yet inspected.

Our third specification is shown in Equation 4.

| $y_{it}=b_{0}+\sum_{k=1}^{4} {b_{1k}[pre}_{it}^{k}]+$  $\sum_{k=1}^{4} {b_{2k}[post\_1}_{it}^{k}]+$  $\sum_{k=1}^{4} {b_{3k}[post\_2}_{it}^{k}]+{b_{4}{size}_{it}+\tau}_{t}+a_{i}+e_{it}$ | 4 |
| --- | --- |

Where $y$, ${post\_1}_{it}^{k}$, $size$, $\tau, a$, and $e$ are defined as above. ${pre}_{it}^{k}$ and ${post\_2}_{it}^{k}$ are two additional sets of variables each measuring the rating score specific change in prescribing behaviour in the pre inspection (1 month) and longer term post inspection (greater than six months) periods. This specification is designed to detect if practices respond in anticipation of an inspection and also how long the post inspection effect might last.

|  |  |
| --- | --- |
